# Impact of the oxaliplatin-5 fluorouracil-folinic acid combination on respective intracellular determinants of drug activity

**DOI:** 10.1038/sj.bjc.6600185

**Published:** 2002-04-08

**Authors:** J L Fischel, P Formento, J Ciccolini, P Rostagno, M C Etienne, J Catalin, G Milano

**Affiliations:** Centre Antoine Lacassagne, Oncopharmacology Unit, 33, Avenue de Valombrose, 06189 Nice Cedex 2, France; School of Pharmacy, Pharmacokinetics Unit, Marseille, France

**Keywords:** 5-fluorouracil, oxaliplatin, drug interactions, WiDr cell line

## Abstract

The combination of 5-fluorouracil-folinic acid and oxaliplatin has led to a significant improvement of chemotherapy efficacy in advanced pretreated colorectal cancer. The objective of the present study was, considering the oxaplatin-5-fluorouracil-folinic acid combination, to examine the impact of one given drug on the cellular determinants of cytotoxic activity of the other drug. These cellular factors were analysed on the human colon cancer cell line WiDr in clinically relevant conditions of drug exposure (‘De Gramont’ schedule) with oxaliplatin-folinic acid during 2 h followed by 5-fluorouracil 48 h. The DNA binding of oxaliplatin was significantly reduced by the presence of 5-fluorouracil but this effect was time-dependent and after 50 h the platinum incorporated into DNA was identical in controls and in the drug combination. In the presence of oxaliplatin, there was less formation of FUH_2_ which is the first catabolite produced in the cascade of 5-fluorouracil metabolic degradation. The effects of drugs on cell cycle were quite different from one drug to the other with oxaliplatin inducing a shift towards G_2_ accumulation and 5-fluorouracil-folinic acid to a greater proportion of cells in G_1_–S. When oxaliplatin and 5-fluorouracil-folinic acid were combined the cell cycle effects were very similar to that of the 5-fluorouracil-folinic acid sequence alone. Oxaliplatin was able to reduce thymidylate synthase activity with a marked impact 28 h after the beginning of cell exposure to the drug. The 5-fluorouracil-folinic acid drug sequence led to a profound reduction in thymidylate synthase activity and this decrease was not markedly enhanced by the presence of oxaliplatin. Regarding apoptosis, changes in mitochondrial membrane permeability were observed in the presence of the tested drugs and the impact of 5-fluorouracil-folinic acid was greater than that of oxaliplatin. The addition of oxaliplatin did not amplify the action of 5-fluorouracil-folinic acid upon mitochondrial membrane permeability change. The presence of oxaliplatin itself did not modify the intracellular concentration of total reduced folates. The fact that oxaliplatin may reduce 5-fluorouracil catabolism could be central in explaining the supra-additive interaction between these drugs.

*British Journal of Cancer* (2002) **86**, 1162–1168. DOI: 10.1038/sj/bjc/6600185 www.bjcancer.com

© 2002 Cancer Research UK

## 

Colorectal cancer remains a leading cause of cancer deaths in western countries and approximately half of all patients develop metastatic disease carrying a poor prognosis. Chemotherapy still represents the treatment of choice for palliative purposes. The efficacy of chemotherapy in advanced colorectal cancer has significantly progressed during the last two decades. Folinic acid (FA) modulation of 5-fluorouracil (FU) has markedly increased the tumour response rate and, more recently, new drugs have emerged in the arena of colorectal cancer treatment. Irinotecan and oxaliplatin (Oxa) are representative of these new active drugs. Irinotecan (CPT 11) is a topoisomerase I inhibitor which prolongs patient survival as a second-line treatment in advanced colorectal cancer resistant to FU (Van Cutsem *et al*, 1999). Oxa displays activity not only in patients with platinum-sensitive malignancies such as ovarian tumours but also in those with colorectal cancer, a disease known for its resistance to both cisplatin and carboplatin (Judson and Kelland, 2000). Recently, combinations of FUFA and irinotecan or of FUFA and Oxa have led to a further improvement of chemotherapy efficacy in advance pretreated colorectal cancer (André *et al*, 1998; Ducreux *et al*, 1999). Importantly, a recent controled trial has established the superiority of Oxa-FUFA over FU FA in terms of response rate and progression-free survival (De Gramont *et al*, 2000). The high antitumour efficacy of this combination protocol is in agreement with preclinical data (Raymond *et al*, 1997) showing the synergistic cytotoxic activity of the Oxa-FUFA association.

The objective of the present study was, when considering the Oxa-FUFA combination, to examine the impact of one given drug on the cellular determinants of cytotoxic activity of the other drug. Different potentially relevant cellular parameters for the activity of combined drugs were analysed on the human colon cancer cell line WiDr in clinically relevant conditions of drug exposure. The tested drug sequence was Oxa-FA during 2 h followed by FU during 48 h. This sequence faithfully matches the currently applied clinical protocol derived from the ‘De Gramont’ schedule (De Gramont *et al*, 2000). The choice of cellular parameters was dictated by the respective mechanisms of action of FU and Oxa. These parameters were: the incorporation of platinum (Pt) in nucleic acids, the levels of thymidylate synthase (TS) activity and TS protein, the intracellular profile of FU metabolism, the incorporation of FU metabolites in nucleic acids, the intracellular levels of reduced folates and cell cycle analysis. In addition, the impact on apoptosis of individual drugs and drug combinations was taken into account. For this latter purpose, change in mitochondrial membrane permeability was examined as it is one of the key events initiating the apoptotic process.

## METHODS

### Chemicals

All the chemicals were obtained from Sigma Chemical Co (St Quentin Fallavier, France) and were of the highest purity available. Regular DMEM and glutamine were obtained from Whittaker (Verviers, Belgium) and foetal bovine serum from Dutscher (Brumath, France). Penicillin and streptomycin were obtained from Merieux (Lyon, France). FU and Oxa were the pharmaceutical forms obtained from Roche (Neuilly/Seine, France) and Sanofi Synthelabo (Gentilly, France), respectively. FA (racemic mixture) was from Sigma Chemical Co.

### Drug administration schedule

The human colorectal cancer cell line, WiDr, was used in the present study. Cells were routinely cultured in DMEM supplemented with 10% foetal bovine serum, 2 mM glutamine, 50 000 units^−1^ penicillin and 80 μM streptomycin in a humidified incubator (Sanyo, Japan) at 37°C with an atmosphere containing 8% CO_2_. One week before commencement of the experiments, the cells were grown in a folate-controlled medium (folic acid-free DMEM supplemented with 40 nM of DL-5 methyltetrahydrofolate and 0.1 mM of L-ascorbate) to simulate as closely as possible the physiological conditions encountered in humans (Kones, 1990). The above folate-controlled medium was used throughout the experiments.

Cells were plated in 96-well microtitration plates (100 μl well^−1^) to obtain exponential growth for the whole duration of the experiment (initial cell density was 2500 cells per well). Twenty-four hours later, cells were exposed to the drugs. Cytotoxic efficacy of drugs taken alone was first considered. This permitted the determination of individual IC_50_ values (MTT test, Carmichael *et al*, 1987). Oxa (at IC_50_=19.5 μM) and d, l FA (10 μM) were applied for 2 h followed by FU during 48 h (at IC_50_=13.5 μM).

The cytotoxic effects resulting from this drug combination were checked in previously described experimental conditions (Fischel *et al*, 1998 unshown experiments). For a drug combination, the calculation of the Chou and Talalay (1984) combination index indicates the resulting effect of the drug association. A combination index at 1.00 means simple additivity, below 1 means synergy and higher than 1 means antagonism. A mean combination index of 0.8 was found in our experiment confirming the existence of slight synergistic (supra-additive) cytotoxic activity (Fischel *et al*, 1998). Cellular parameters were analysed between 2 and 98 h after the onset of different drug applications. Treated and control cells were collected at different sampling times (2, 8, 28, 50, 74, 98 h after the onset of Oxa applications). All investigations were duplicated at a distance.

### Cell cycle analysis

The cell cycle was analysed by FACS according to the Vindelov and Christensen (1990) method. The percentage of cells in different cell cycle phases was determined by Modfit software.

### Evaluation of mitochondrial membrane permeability

Cells were treated with or without various concentrations of drugs as described above. At various intervals, cells were washed with PBS, trypsinised and then placed in DMEM medium.

The measuring of ΔΨm was performed with a cationic lipophilic fluorochrome: 3,3′ dihexyloxacarbocyanine iodide (DiOC_6_(3)) purchased from Sigma. Cells were incubated at 37°C for 15 min in the presence of DiCO_6_(3) 40 nM, then placed on ice followed by addition of propidium iodide 10 μg ml^−1^. The fluorochrome incorporations were immediately analysed using a Becton Dickinson FACScan flow cytometer at an excitation wavelength of 488 nm. PI was used in all samples to exclude dead cells from the analysis. DiOC_6_(3) fluorescence was recorded in FL1 and PI was recorded in FL3. The percentage of apoptosis cells was calculated from an FL1/FL3 scattergram. All experiments were repeated three times.

### TS measurements and reduced folate determination

- TS activity was measured according to the tritium-release assay described by Spears and Gustavsson (1988). The assay consisted in incubating 25 μL of cytosol with ^3^H-dUMP (1 μM final concentration) and 5,10-methylenetetrahydrofolate CH_2_FH_4_, (0.62 mM final concentration) in a total volume of 55 μL. After 0 (for blank substraction), 10, 20, and 30 min of incubation at 37°C, the reaction was stopped in ice. The excess of ^3^H-dUMP was removed by adding 300 μL of activated charcoal (15%) containing 4% trichloroacetic acid (5-min centrifugation at 14 000 **g**, room temperature). The ^3^H_2_O formed during the incubation was then counted in an aliquot of the above supernatant. Results were expressed as fmoles of ^3^H_2_O formed per min per mg of protein, based on the linear regression obtained from the incubation times. The sensitivity limit was 10 fmol min^−1^ mg^−1^ prot. Inter-assay reproducibility was evaluated through repeated analysis of single-use aliquots of a pooled cytosol: *n*=5, mean=1110 fmol min^−1^ mg^−1^ prot, s.d.=78.59 fmol min^−1^ mg^−1^ prot, CV=7.08%.
- Concentration of total reduced folates (CH_2_FH_4_+FH_4_) and total TS protein were measured with the radioenzymatic method previously described by Bunni *et al* (1988). The ternary stable complex ^3^HFdUMP-TS-CH_2_FH_4_ was used as a trap for the measurement of either TS or total reduced folates:
- Measurement of TS protein with excess of ^3^HFdUMP and CH_2_FH_4_.
- Measurement of total reduced folates with excess of ^3^HFdUMP and TS (presence of formaldehyde allowing the conversion of FH_4_ into CH_2_FH_4_).
- The excess of radioactivity was retained on Sephadex columns and the eluted fraction was counted by liquid scintillation. The sensitivity limit for TS was 0.010 pmol mg^−1^ prot with a CV=15%. The sensitivity limit for total reduced folates was 0.3 pmoles mg^−1^ prot with a CV=10%.

### Drug incorporations in nucleic acids

Platinum incorporation into DNA was measured as follows: after isolation of DNA on Qiagen® columns by ionic strength elution, DNA platinum incorporation was measured using Atomic Absorption Spectrophotometry.

^14^C FU incorporation into RNA and DNA was determined as follows: WiDr cells growing exponentially in 25 cm^2^ flasks were put in contact at different exposure times –8, 28, 50, 74, 96 h with ^3^H FU (13.5 μM, specific activity 56 mCi mMole^−1^) with or without FA (10 μM) and Oxa (19.6 μM) DNA and RNA were isolated on Qiagen® columns. Radioactivity incorporation was evaluated on a liquid scintillation counter.

### Analysis of intracellular metabolism of 5-FU

^3^H FU metabolites were separated by HPLC and counted on line by radioactivity monitoring as described previously (Ciccolini *et al*, 2000). Exponentially growing cells were exposed to various combination of 100 μCi of tritiated FU (final concentration 2 μM). Cells were harvested and cytosols were isolated for HPLC analysis. The HPLC consisted of an HP 1090 (Hewlett Packard) system coupled with an A 200 radioactive flow detector (Packard). Separation of tritiated metabolites was achieved using a Lichrospher 100 RP 18 5 μM column (Hewlett Packard) eluted by 50 mM K_2_HPO_4_ (pH 6.8) containing 1 mM tetrabutyl ammonium nitrate and 12% (0–9 min) to 16% (9–60 min) methanol.

### Statistical analysis

**Figure 1:**
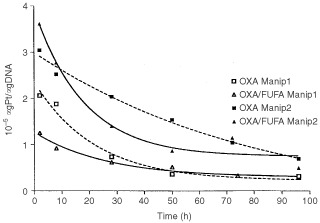
Time course of Pt concentration in DNA for the WiDr cell line (as % of maximum incorporation: 2 h exposure Oxa alone (two independent experiments). Solid and open squares and dashed line: Oxa alone. Solid and open triangles and solid line: Oxa+FUFA. The Pt incorporations are statistically different between the two conditions Oxa *vs* Oxa+FUFA. *P*=0.0055 (paired *t*-test on all shown data).

**Figure 2:**
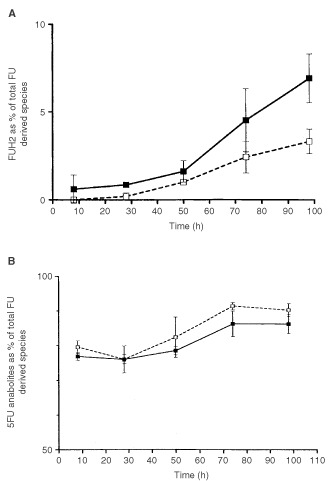
(**A**) Time course of FUH2 concentration (as % of total FU derived chemical species). Solid square and solid line: FUFA alone. Open square and dashed line: FUFA+Oxa. Bars represent Standard Deviations (two independent experiments). The two curves are statistically different *P*=0.0042 (paired *t*-test). (**B**) Time course of anabolites concentration (sum of FUrd, FUMP, FUDP, FUTP, FdUrd, FdUMP, FdUDP, FdUTP). Solid square and solid line: FUFA alone. Open square and dashed line: FUFA+Oxa. Bars represent Standard Deviations (two independent experiments). The two curves are statistically different *P*=0.0096 (paired *t*-test).

**Figure 3:**
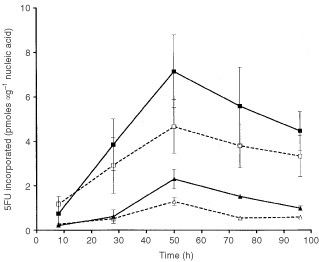
Time course incorporation of ^14^CFU in nucleic acids. Solid square and solid line: DNA, FUFA alone. Open square and dashed line: DNA, FUFA+Oxa. Solid triangle and solid line: RNA, FUFA alone. Open triangle and dashed line: RNA, FUFA+Oxa. Bars represent Standard Deviations (two independent experiments). The two curves of RNA incorporation are statistically different *P*=0.0066. The two curves of DNA incorporation are statistically different *P*=0.0.156 (paired *t*-test).

Comparisons between different tested conditions were done with Wilcoxon non parametric paired tests for experiments described in Figures 1, 2A,B and 3. Comparisons between different tested conditions were done with Mann-Whitney unpaired non parametric test for experiments described in Figures 5A,B and 6.

## RESULTS

Figure 1

depicts the impact of the presence of FU on the incorporation of Pt into DNA. Although not superimposable due to experiments performed at distance, the results shown in Figure 1 point out that DNA binding of Oxa was significantly reduced by the presence of FU but this effect was time-dependent and after 50 h the Pt incorporated into DNA was identical in controls (Oxa alone) and in the drug combination (Oxa FUFA sequence).

The presence of Oxa was found to modify the intracellular metabolic profile of FU. Under Oxa, there was less formation of FUH_2_ (Figure 2A

), which is the first catabolite produced in the cascade of FU metabolic degradation. Conversely, more FU anabolites were formed in the presence of Oxa (Figure 2B). When considering the global incorporation of ^14^CFU into nucleic acids, and contrary to expectations, there were fewer FU-related compounds present in nucleic acids following the Oxa FUFA sequence as compared to FUFA alone (Figure 3

).

The respective impacts of Oxa and Oxa_FUFA on cell cycle organisation are detailed in Table 1

**Table 1:**
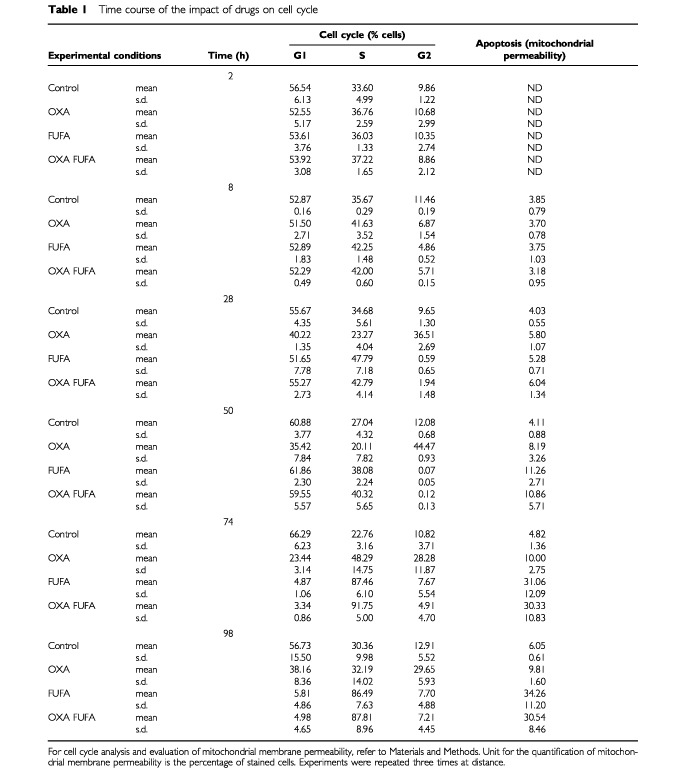
Time course of the impact of drugs on cell cycle

. These effects on cell cycle are quite different from one drug to the other with Oxa inducing a shift towards G_2_ accumulation and FUFA leading to a greater proportion of cell in G_1_–S and a lower proportion in G_2_ phase. It is interesting to note that when Oxa and FUFA were combined the cell cycle effects were very similar to that of the FUFA sequence alone (Table 1).

**Figure 4:**
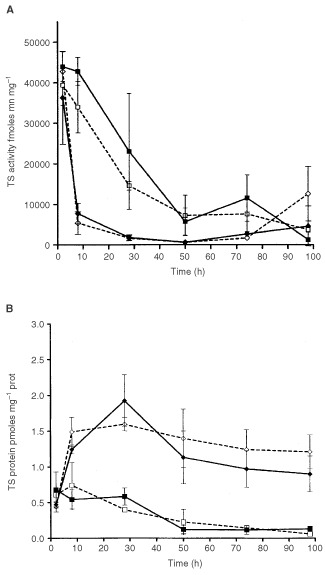
(**A**) Time course of TS activity. Solid square and solid line: Control without drugs. Open square and dashed line: Oxa alone. Solid diamond and solid line: FUFA alone. Open diamond and dashed line: FUFA+Oxa. Bars represent Standard Deviations (two independent experiments). The curves Control and Oxa alone do not differ significantly. FUFA alone and FUFA+Oxa do not differ significantly. Control and FUFA alone differ significantly *P*=0.0163. Oxa alone and FUFA+Oxa differ significantly *P*=0.0034. Non parametric unpaired test. (**B**) Time course of TS protein. Solid square and solid line: Control without drugs. Open square and dashed line: Oxa alone. Solid diamond and solid line: FUFA alone. Open diamond and dashed line: FUFA+Oxa. Bars represent Standard Deviations (two independent experiments). The two curves control and Oxa alone do not differ significantly. FUFA alone and FUFA+Oxa do not differ significantly. Control and FUFA alone differ significantly *P*<0.01. Oxa alone and FUFA+Oxa differ significantly *P*<0.001.

The modifications in TS activity and TS protein levels under drug applications are shown in Figure 4A

and B respectively. This reduction in TS activity of the control cells may be attributable to progressive cellular confluence in the small well plates. It appears that Oxa alone is able to reduce TS activity with a marked impact 28 h after the beginning of cell exposure to the drug. The FUFA drug sequence leads to a profound reduction in TS activity. Although this decrease is enhanced in its early phase (8 h) by the presence of Oxa (Figure 4A) the ‘FUFA alone’ curve and the ‘FUFA+OXA’ curve are almost identical. An early, marked and sustained rebound in TS protein is observed after cell exposure to FUFA. This rebound was found to be maintained in the presence of Oxa (Figure 4B).

Changes in mitochondrial membrane permeability were observed in the presence of the tested drugs. These effects are detailed in Table 1. The impact of FUFA is greater than that of Oxa. The maximal effect of Oxa is observed at 74 h whereas FUFA has maximal impact at 98 h. Globally, the addition of Oxa does not amplify the action of FUFA upon mitochondrial membrane permeability change.

**Figure 5:**
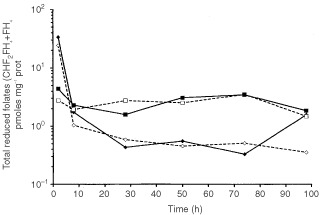
Time course of total reduced folates concentration (logarithmic scale, s.d. varied between 0.009 and 1.94). Solid square and solid line: control without drugs. Open square and dashed line: Oxa alone. Solid diamond and solid line: FUFA alone. Open diamond and dashed line: FUFA+Oxa. The two curves: control and Oxa alone are not statistically different. FUFA alone and FUFA+Oxa are not statistically different. Control and FUFA alone are statistically different *P*=0.0469. Oxa alone and FUFA+Oxa are statistically different *P*=0.039.

The intracellular evolution of total reduced folates is shown in Figure 5

. The initial decrease in total reduced folates is due to the changing of the medium from 10 μM FA during the first 2 h of drug exposure to normal medium conditions. The presence of Oxa itself does not modify the intracellular concentration of reduced folates. Noteworthy, it was found that a marked decrease in reduced folate levels occurred in the presence of FUFA. This time profile of intracellular reduced folates was very similar to that observed when adding Oxa to FUFA (Figure 5).

## DISCUSSION

The objective of the present study was, when considering the Oxa-FUFA combination, to examine the impact of one given drug on the cellular determinants of cytotoxic activity of the other drug. To that end, the individual cellular targets of the considered drugs were examined. The clinically relevant drug sequence consisting in Oxa-FA, 2 h followed by FU, 48 h (De Gramont *et al*, 2000), was adopted in the present study. The supra-additive interaction which had previously been shown (Fischel *et al*, 1998) was confirmed in the present study on the WiDr human colon cancer cell line using the same conditions (drugs, concentration, time of exposure) used for the previously published results. The presence of cytotoxic synergy between Oxa and FU had been previously reported by Raymond *et al* (1997) with both *in vitro* and *in vivo* data on human HT 29 colon cancer xenografts. In this latter study, examining possible explanatory factors, the authors failed to show that FU could significantly modify the tumoural total Pt DNA binding of Oxa during Oxa-FUFA combination treatment. In the present study, it was shown that the addition of FU to OXA resulted in a decrease of Pt binding to DNA (Figure 1). However, this reduction of Pt binding is moderate and one of the possible reasons for this phenomenon, which occurs during cell exposure to FU, may lie in the fact that FU anabolites can be fraudulously incorporated into RNA and DNA (Grem, 2000) and this incorporation may compromise the binding of Pt to nucleic acids. Conversely, and probably for the same reasons of reciprocal impairment of nucleic acid drug binding, it was found that, during cell exposure to Oxa-FUFA, there were fewer FU anabolites incorporated into nucleic acids as compared to what is observed with the FUFA sequence alone (Figure 3).

In the present study, using flow cytometry analysis, it was shown that the FUFA sequence induces an accumulation of cells in the G_1_ early S phase. This observation is in agreement with known comparable data (Pizzorno *et al*, 1995) recently confirmed by Backus *et al* (2001). The impact of Oxa on cell cycle with an increased proportion of cells in the G_2_ phase also concurs with the cell cycle effects of platinum derivatives (Mastbergen *et al*, 2000). Interestingly, after Oxa and FUFA combination, the organisation of cells within the cellular cycle (G_1_–S shift) was superimposable to that observed following FUFA alone (Table 1). This accumulation of cells in S phase is compatible with a slow-down of cell proliferation in agreement with the decrease in TS activity which was observed in the present study. Indeed, cell proliferation and TS activity are closely related as recently underlined by the data of Grem *et al* (2001).

The enzyme dihydropyrimidine dehydrogenase (DPD) regulates the FU catabolic route in liver and, importantly, at the tumoural cell level (Beck *et al*, 1994). The impact of the variability in tumoural DPD activity on FU cytotoxic activity has been previously demonstrated with both *in vitro* (Fischel *et al*, 1995) and *in vivo* data (Etienne *et al*, 1995). This resulted in the recent development of DPD inhibitors aimed at optimising FU-based chemotherapy (Hoff and Pazdur, 1999; Diasio, 2000). DPD activity was previously thought to be inhibited by cisplatin, a clinically-proven FU modulator (Leteurtre *et al*, 1990; Nishiyama *et al*, 1999). Recent pharmacokinetic studies have suggested that Oxa may inhibit FU catabolism (Papamichael *et al*, 1998), although this finding was not confirmed by other investigators (Graham *et al*, 2000). The present investigation included a close examination of the main FU metabolic routes. At this level, it appears that in the presence of Oxa there was a significant decrease in the intracellular levels of FUH_2_. This points to the possibility that Oxa may inhibit DPD activity. Although a direct impact on DPD activity by Oxa was not tested in the present study, this potential DPD inhibition may be of prime importance for tumours which overexpress DPD since it has been shown that DPD-related resistance may be circumvented by the use of DPD inhibitors (Fischel *et al*, 1995). In this view, it is interesting to emphasise that new clinical responses have been observed when adding Oxa to FUFA in colorectal cancer patients resistant to FUFA (André *et al*, 1998; Garufi *et al*, 2000). The shift towards fewer catabolites and more anabolites of FU in the presence of Oxa was not however translated in the present study by an increase in FU anabolite incorporation into nucleic acids (Figure 3). The relative excess of the FU anabolite pool in the presence of Oxa may be at the origin of the more pronounced TS inhibition in the presence of Oxa FUFA (Figure 5A) but globally the impact of FUFA or OxaFUFA on TS activity was superimposable. Interestingly, it was also demonstrated that Oxa itself is able to inhibit TS activity (Figure 5A) but this effect occurs later than the initial marked depletion in TS activity already induced by FUFA. This direct impact of Oxa on TS activity should be kept in mind and further explored in preclinical investigations or clinical studies with different Oxa-FUFA schedules. Other authors have pointed out an effect of Oxa on TS with a smaller FU-induced TSmRNA increase when Oxa is added to Fu (Plasencia *et al*, 2001). In contrast, the present data did not show that the presence of Oxa modified the FU-induced TS protein increase which had been previously described by Chu *et al* (1993).

Optimal inhibition of TS by FU required the formation of a ternary complex between TS, fluorodeoxyuridine monophosphate (a key anabolite of FU) and a reduced folate, CH_2_HF_4_ (Keyomarsi and Moran, 1988). Experimental studies have established that the stabilisation of the ternary complex is enhanced by the presence of high levels of CH_2_FH_4_ (Moran and Scanlon, 1991). On this basis, clinical data have been provided that show better antitumour efficacy when FU is combined with leucovorin (folinic acid), a precursor of CH_2_FH_4_ (Etienne *et al*, 1996). We reported, in cancer patients treated by FU-based chemotherapy, that, at pretreatment stage, the tumoural concentration of reduced folates was a predictor of an objective response to treatment (Etienne *et al*, 1995). Cisplatin has been previously shown to upregulate the intracellular concentration of reduced folates (Vitols *et al*, 1987). We thus examined the possible impact of Oxa on the intracellular levels of total reduced folates. It was found that, in comparison to FUFA, the Oxa-FUFA sequence did not significantly modify the fate of the intracellular profile of reduced folates. The present study included an examination of drug-induced apoptotic events by considering the key step of apoptosis at the mitochondrial level. Both Oxa and FUFA were able to alter mitochondrial permeability with time-modulated effects (Table 1). It must be underlined that FUFA was more active than Oxa and that the addition of Oxa to FUFA did not change the impact on mitochondrial permeability already generated by FUFA. Other apoptotic pathways, like FAS-induced apoptosis, should be studied more closely as this pathway has been recently shown to play a major role in FU-mediated toxicity (Petak *et al*, 2000).

The present study examined the effects on respective cellular determinants of drug activity when combining Oxa and FUFA. The investigations covered the impact on drug targets, drug metabolism, cell cycle effect and apoptosis. Among the various factors explored, many failed to provide objective mechanistic explanations for the synergistic interaction between Oxa and FUFA in particular nucleic acid binding of respective drugs and intracellular concentration in reduced folates. In our opinion, the fact that Oxa may reduce FU catabolism at the DPD level could be central in explaining the supra-additive interaction between Oxa and FUFA. According to this hypothesis a DPD inhibitor could reduce the synergy of the Oxa FUFA combination. This must be kept in mind for further combinations between Oxa and recently-developed FU oral prodrugs containing a DPD inhibitor. It is possible that other molecular mechanisms may contribute to the supra-additive cytotoxic effects of the Oxa-FUFA combination. The present data may provide useful rational grounds for discussion when testing clinical confirmation of the impact of Oxa on FU catabolism. In this framework, exploring lymphocytic and tumoural DPD activity under Oxa treatment seems relevant.
